# Highly Water-Absorptive and Antibacterial Hydrogel Dressings for Rapid Postoperative Detumescence

**DOI:** 10.3389/fbioe.2022.845345

**Published:** 2022-05-13

**Authors:** Yuan Fang, Haibo Li, Jingting Chen, Yao Xiong, Xu Li, Jianda Zhou, Shengli Li, Shoubao Wang, Binbin Sun

**Affiliations:** ^1^ Department of Plastic and Reconstructive Surgery, Shanghai Ninth People’s Hospital, Shanghai Jiao Tong University School of Medicine, Shanghai, China; ^2^ Department of Plastic Surgery, The Third Xiangya Hospital, Central South University, Changsha, China; ^3^ Department of Orthopaedics, Shanghai Ninth People’s Hospital, Shanghai Jiao Tong University School of Medicine, Shanghai, China; ^4^ Collage of Biological Science and Medical Engineering, Donghua University, Shanghai, China

**Keywords:** hydrogel, detumescence, antibacterial, MgSO_4_, MgO

## Abstract

Postoperative wound edema, infection, and pain burden the patient’s life. Therefore, the purpose of this study is to develop an effective antibacterial, multifunctional application to prevent postoperative edema and relieve postoperative pain by making full use of the dehydrating and analgesic effects of magnesium sulfate (MgSO_4_), magnesium oxide (MgO), sodium alginate (SA), and sodium carboxymethyl cellulose (Na-CMC) to make a composite hydrogel, which can promote postoperative detumescence. MgSO_4/_/MgO/SA/Na-CMC composite hydrogel dressings have outstanding mechanical properties, high water absorption, and good biocompatibility. MgO endows the hydrogel dressing with excellent antibacterial properties and better antibacterial activity against common bacteria and multidrug-resistant bacteria. In addition, MgSO_4_/MgO/SA/Na-CMC hydrogel dressing shows superior dehydration and analgesic properties in the postoperative nude mice model. This study shows that the multifunctional MgSO_4_/MgO/SA/Na-CMC composite hydrogel dressing developed as a surgical incision dressing has broad prospects in the prevention of incision infection, postoperative edema, and analgesia.

## Introduction

A postoperative incision is usually accompanied by complications such as edema and infection, which are significant problems for patients and medical staff (Maheshwer et al., 2021). Especially in plastic surgery, patients have obvious facial edema after the operation, which significantly affects their life and work. Effective incision management after the operation can not only reduce complications and human and material costs but also achieve rapid recovery and reduce unnecessary troubles to life and work caused by surgical complications (El Hosary et al., 2020).

Because of their soft texture and viscosity, hydrogels have been very popular with researchers in recent years (Singh et al., 2013; Bayer, 2021; Cheng et al., 2021; Guimaraes et al., 2021). Hydrogels are ideal dressings with biocompatibility. At the same time, it can be used and replaced according to the needs of patients. This not only reduces the inconvenience of repeated medical treatment after an operation but also reduces the workload of doctors. Because hydrogels are more commonly used as postoperative dressings, at present, many studies reported that hydrogel dressings can achieve bacteriostatic effects or provide the best pH value to promote wound healing (Cheng et al., 2021; Fan et al., 2021; Liang et al., 2021). At present, the clinical hydrogel material used for postoperative incision edema is “cold compress.” The principle of cold compress is only to promote vasoconstriction and reduce postoperative swelling. This solution has obvious limitations: first, only relying on physical cooling to reduce swelling has a limited effect; and second, the postoperative incision is still at risk of infection. Few dressings focus on the needs of plastic surgery for detumescence, anti-infection, and pain. To avoid postoperative edema and infection, the hydrogel dressings we studied should have good water absorption and antibacterial ability.

It is well known that oral magnesium sulfate (MgSO_4_) forms a hypertonic environment in the intestine and has the function of catharsis (Krenzelok et al., 1985). Magnesium oxide (MgO) also has an antibacterial effect (Muniz Diaz et al., 2021; Wang et al., 2021). Although some researchers have added antibiotics to the dressing to achieve antibacterial effect, it will lead to bacterial resistance (Fischbach and Walsh, 2009). We may solve this issue by directly including MgO, a material with antibacterial activity, into the dressing. At present, hydrogel technology based on sodium alginate (SA) and sodium carboxymethyl cellulose (Na-CMC) has been very mature, which can carry drugs for the treatment of related diseases (Sheng et al., 2021). Therefore, it is feasible for us to add MgSO_4_/MgO into hydrogels. In this dressing, we used MgSO_4_ with water absorption and MgO with antibacterial effect. According to the already mature SA/Na-CMC hydrogel preparation technology, combined with MgSO_4_ and MgO, a new dressing was prepared. This dressing had both absorptive and antibacterial hydrogel dressing and could accelerate wound detumescence after operation. It is very valuable in clinical application.

To this end, we mixed MgSO_4_, MgO, SA, and Na-CMC in a certain proportion to prepare hydrogels with high water absorption and antibacterial effects. We evaluated the appearance of MgSO_4_/MgO/SA/Na-CMC composite hydrogel dressings by gross characterization. The internal structure of the hydrogels was examined by scanning electron microscopy. The swelling ratio of the hydrogels was used to assess the water absorbency of hydrogel dressings. After HaCaT cells were co-cultured with the hydrogel extract for a period of time, the biocompatibility of the hydrogel dressing was evaluated by live/dead staining. The antibacterial activity of hydrogel dressings was tested by bacteriostasis experiments of *Escherichia coli* and *Staphylococcus aureus*. The surgical incision edema model was used to detect the swelling effect of hydrogels *in vivo*.

## Materials and Methods

### Materials

Sodium alginate (SA), magnesium sulfate (MgSO_4_), magnesium oxide (MgO), sodium carboxymethyl cellulose (Na-CMC), and glycerol were purchased from Shanghai Aladdin Biochemical Technology Co., Ltd.

### Hydrogel Preparation

We used the dosage of materials listed in Table 1 and added each material to prepare the composite hydrogels. At room temperature, 1 ml glycerol was added to 9 ml double-distilled H_2_O (dd H_2_O). At 25°C, MgSO_4_ and MgO (MgSO_4_: MgO = 99:1 w/w) powders of different weights were separately added to the aforementioned solution and stirred for 30 min. After adding SA and Na-CMC successively, the solution was stirred in a 65°C constant temperature water bath for 6 h. The thicker liquid was added to the abrasives and placed in the 4°C refrigerator for preservation in subsequent experiments.

**TABLE 1:** The composition list of MgSO_4_/MgO/SA/Na‐CMC composite hydrogels.

	Ctrl	Mg10	Mg20	Mg30	Mg40
ddH_2_O (ml)	9.0	9.0	9.0	9.0	9.0
Glycerol (ml)	1.0	1.0	1.0	1.0	1.0
Mg (%)	0.0	10.0	20.0	30.0	40.0
SA (g)	0.5	0.5	0.5	0.5	0.5
Na-CMC (g)	0.5	0.5	0.5	0.5	0.5

### Morphological Assay

The appearance of these five groups of hydrogels was photographed with digital cameras. The prepared MgSO_4_/MgO/SA/Na-CMC hydrogel dressings were freeze-dried for 48 h at −80°C. The samples were cut with a small blade and placed on the scaffold. The internal morphology of the hydrogels was observed by scanning electron microscopy (SEM).

### Mechanical Properties

The mechanical properties of these five groups of hydrogels were tested using the HY-940FS universal testing machine (Shanghai, China) at room temperature. The prepared MgSO_4_/MgO/SA/Na-CMC hydrogel dressings were compressed to 50% deformation under wet conditions. The strain–stress curve was obtained and the compression modulus was calculated.

### Swelling Rate Determination

The purpose of the swelling test is to evaluate the ability of materials to absorb solvents. We used ddH_2_O as the experimental solvent. We calculated the swelling rate in the dry and wet states. First, the prepared hydrogel block (d = 1 cm) was freeze-dried at −80°C for 48 h. The weight of the freeze-dried sample (W_0_) was measured. Then, the gel was soaked in 25°C ddH_2_O and weighed once every 5 min. Before weighing, the excess water was removed from the sample surface with a filter paper, and the recorded value was W_t_. This was continued until the weight remained constant. Second, the swelling capacity of wet hydrogels is measured. The back skin of mice was intercepted. The prepared hydrogels with a diameter of 1 cm were weighed and recorded as W_0_. Mice skin was placed on the cell strainer (biosharp, BS-100-CS), infiltrated in ddH_2_O, and five groups of hydrogels were placed on the top of each group, with three repeated experiments in each group. The weight W_t_ was recorded every 2 h until the weight was constant.

The swelling rate (SR) was calculated by the following formula:

$$SR\left(\%\right)=\frac{W_{t}-W_{0}}{W_{0}}\times 100\%.$$

Among them, (W_t_) is the weight of the swelling hydrogel at the time (T) and (W_0_) is the initial weight of the sample. For each group of three samples, the average value and mean square error are calculated to minimize the error.

### Biocompatibility

The synthesized epidermal cells (HaCaT cells) were cultivated in the hydrogel extract, and the state of the cells was determined using live/dead staining assay to assess the hydrogel’s biocompatibility. The HaCaT cells were inoculated in 24-well plates and incubated in a humidified incubator at 37°C and 5% CO^2^ for three days. The sterilized hydrogels were soaked in sterile PBS for 48 h. Then, 100 ul of the net extract soaked for 48 h was absorbed and co-cultured with 900 ul medium for 24 h. Then, 100 ul of the net extract soaked for 48 h was absorbed, and the cells were co-cultured with 900 ul medium for 48 h. The stained cells were observed under a fluorescence microscope. Living cells absorb calcein AM and emit green fluorescence, while PI enters dead cells and emits red fluorescence after binding with DNA.

### Antibacterial Activity Evaluation

We used common *Escherichia coli* (ATCC 8739) and *Staphylococcus aureus* (ATCC 6538) to carry out the antibacterial activity on the surface of the hydrogel dressing. The hydrogel dressings were placed on an agar plate (petri dish) and incubated for 72 h at 37°C The diameter of the bacteriostasis circle on the agar plate (petri dish) was calculated. Each cycle was repeated three times and the average value was calculated.

### Animals

Male BALB/c nude mice aged 6–8 weeks were purchased from Shanghai Jie SiJieLaboratory Animal Co., Ltd. All animals involved were treated according to the protocol evaluated and approved by the Ethics Committee of the Ninth People’s Hospital, affiliated to Shanghai Jiao Tong University School of Medicine. The mice were randomly divided into five groups with three mice in each group. The experiment was repeated with an intraperitoneal injection of anesthesia. Normal saline containing DAPI fluorescence staining was injected into the subcutaneous soft tissue. After soft tissue swelling of the extremities, the prepared hydrogel was clung to the skin surface. Hydrogels were extracted 4–6 h later. A fluorescent microscope was used to observe whether there was fluorescence in the hydrogel. By observing the swelling of the extremities in nude mice, we could see if the hydrogel was effective.

### Statistical Analysis

All experiments had three groups of repeated control, and the mean ± SD data (*n* = 3) were expressed. The analysis was performed by ANOVA (one-way analysis of variance) test. Student’s t-test was used to compare the two groups. *P* ≤ 0.05 was considered statistically significant.

## Results and Discussion

### Characterization of Prepared Hydrogels

**FIGURE 1:**
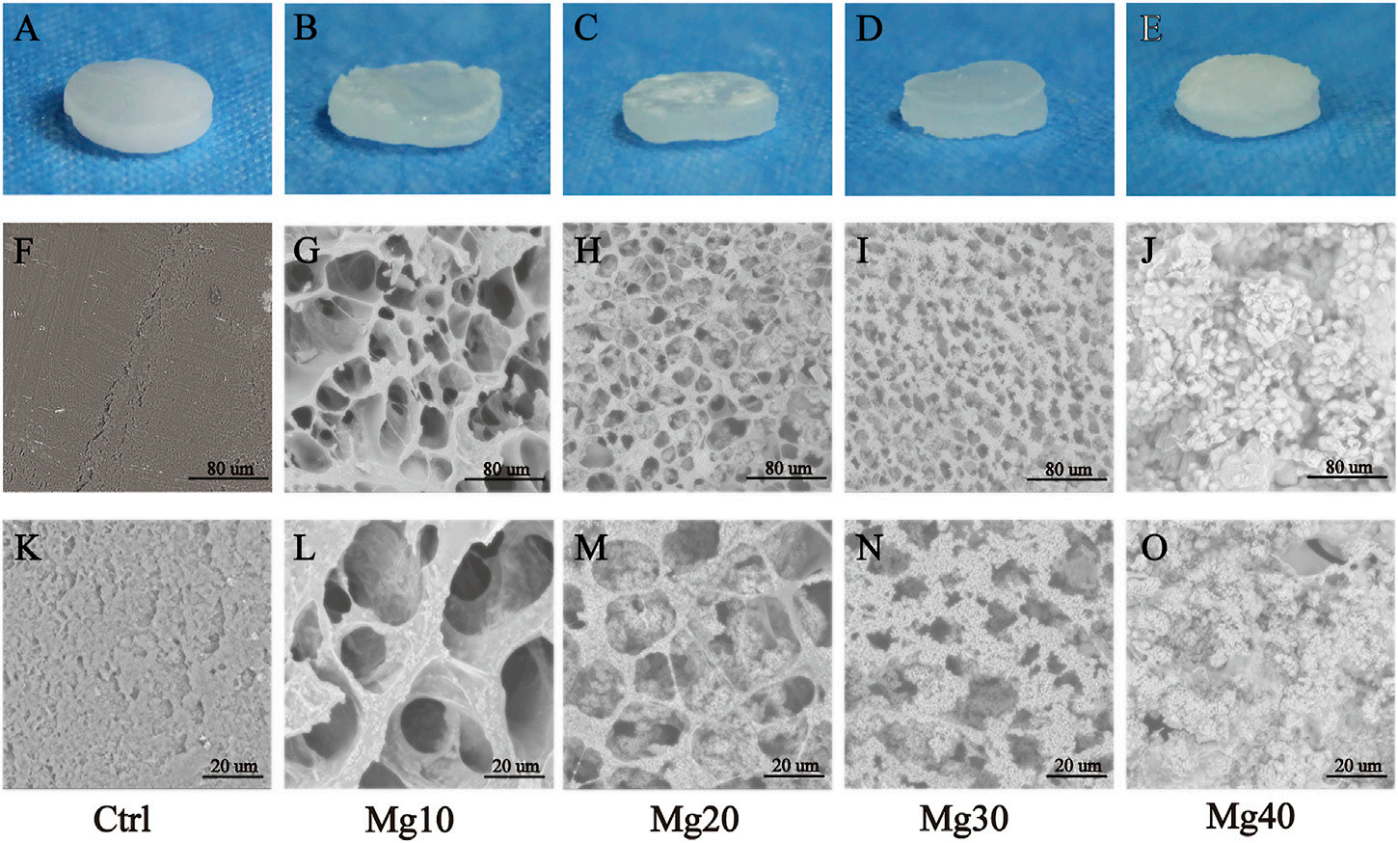
**(A–E)** The appearance of MgSO_4_/MgO/SA/Na-CMC composite hydrogel dressings. SEM images of the internal structure of hydrogels with low **(F–J)** and high **(K–O)** magnification.

The formulations of these five groups of hydrogels (Ctrl, Mg10, Mg20, Mg30, and Mg40) are shown in Table 1. MgSO_4_/MgO/SA/Na-CMC hydrogels were successfully prepared with different MgSO_4_ ratios. The visual examination of hydrogels showed an opaque or translucent appearance, while pure hydrogels without Mg were white (Figures 1A–E). The hydrogels were homogenous and soft, with a diameter of 1 cm and a thickness of about 2–3 mm.

Scanning electron microscopy (SEM) was used to examine the morphology of freeze-dried hydrogels. Figures 1F–O show the internal morphology of these five groups of MgSO_4_/MgO/SA/Na-CMC hydrogels. The results showed that the pure hydrogel material (Ctrl group) was unable to maintain the porous structure because of the shrinkage of the material during the freeze-drying process (Figures 1F,K), while the addition of MgSO_4_/MgO effectively maintained the porous structure of the hydrogel, which showed an interconnected and homogenous porous structure, and the pore size was about 20–100 μm (Figures 1G,L). However, with the increase of magnesium sulfate content, many magnesium sulfate crystals were evenly dispersed in the pores and the pore size was reduced to 20 μm approximately (Figures 1H,L–N). In Figures 1J, O the pore structures are almost invisible because most of them are filled with magnesium sulfate crystals. In Supplementary Figure S1, it is shown that the average pore size of groups Mg10, Mg20, and Mg30 was 34.30 ± 2.13 um, 21.32 ± 0.83 um, and 13.17 ± 0.39 um, respectively (Mean ± SEM). It indicated that the pore size decreased with the increase of the magnesium compound content of the hydrogel. Generally, a smaller pore size can improve the porosity of the hydrogel, enhancing its absorption. In addition, the mechanical properties of the five groups of hydrogels were tested and shown in Supplementary Figure S2. The compression modulus of the five groups was decreased with more magnesium compounds. This may be due to the increase of magnesium compounds, which improves its porosity but reduces its material density; hence, it reduces its mechanical properties. We found that the mechanical properties of the group Mg40 were the worst, which may limit its application as dressing.

The past ten years have witnessed the rapid development of hydrogels. The good biocompatibility and high expansion of hydrogels have attracted the attention of many researchers (Pooresmaeil et al., 2019; Silva et al., 2019). SA is water-soluble, can effectively absorb wound secretions, and keeps the wound clean and dry, which is conducive to wound healing. In addition, SA is low-cost and easy to obtain (Gao et al., 2020). At room temperature, calcium ions (Ca^2+^) were crosslinked with SA to form hydrogels to increase mechanical properties (Paudyal et al., 2013). Moreover, Ca^2+^ after crosslinking could help trigger the coagulation mechanism and promote hemostasis when they were released into the wound (Afjoul et al., 2020).

### Swelling Ratio

**FIGURE 2:**
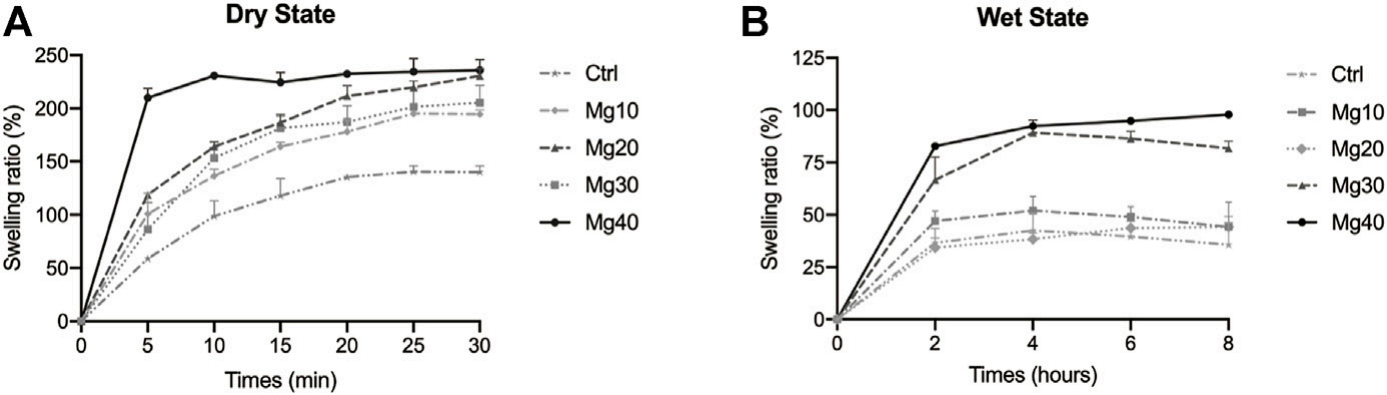
The swelling ratio of different MgSO_4_/MgO/SA/Na-CMC composite hydrogel dresssings in the dry state **(A)** and in the wet state **(B)**.

The higher the SR value, the stronger the water absorbability of the hydrogels will be. This means that the hydrogen absorbs the exudate from the wound more strongly, thus achieving our goal of promoting incision detumescence. Figure 2A shows the percentage of swelling in the dry state containing different concentrations of Mg. The SR was sorted as Mg40 > Mg20 > Mg30 > Mg10 > Ctrl. All hydrogels showed swelling ability, and Mg40 or Mg20 exhibited a higher SR. As shown in Figure 2B, the percentage of swelling in the wet state containing different concentrations of Mg can be observed. The SR was sorted as Mg40 > Mg30 > Mg10 > Mg20 > Ctrl. The SR of the wet hydrogel was weaker than that of the dry state, but the swelling rate of Mg40 and Mg30 was still high. This was mainly due to the hypertonic effect of MgSO_4_, which was consistent with our expectations.

MgSO_4_ had high water absorption and is often used for catharsis (Snyder et al., 2014). We used the hypertonic effect of magnesium sulfate to solve the problem of postoperative incision edema. To select the appropriate concentration of MgSO_4_, we set different MgSO_4_ concentration gradients. Based on a comparison of water absorption in different states, we concluded that the higher the concentration, the better the water absorption effect, which corresponded to the characteristics of MgSO4’s hyperosmotic action (Holland et al., 1960).

### Biocompatibility

**FIGURE 3:**
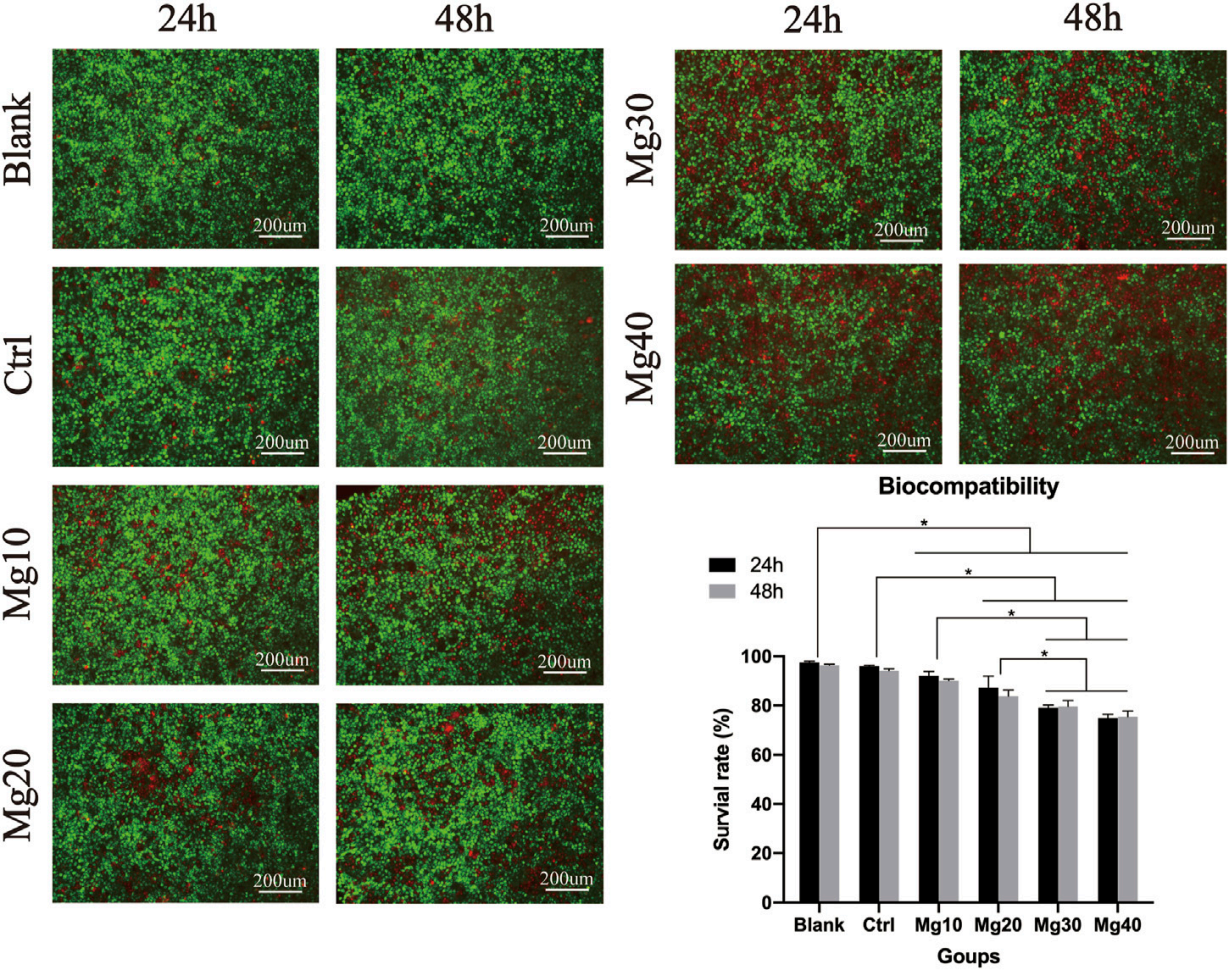
Biocompatibility. P ≤ 0.05 was considered statistically significant (*).

Biocompatibility can help us identify the safety of hydrogels. Five groups of hydrogel extracts were used to culture HaCaT cells for 24 and 48 h to observe the biocompatibility of the hydrogels. After 24 h, the live/dead staining assay showed that there were many living cells (Figure 3). After 48 h of culture, the live/dead staining assay showed that the Mg40 hydrogel contained more than 76% dead cells than other groups. The result of fluorescence staining showed that the hydrogel had good biocompatibility and could be used for biomedical purposes.

Because the hydrogel was closely related to the epidermis, we needed to detect whether it had good biocompatibility using live/death staining detection. Mg^2+^ is often used with biopharmaceuticals because of its good biocompatibility (Gao et al., 2021). As Mg^2+^ is a metal ion, it can inhibit cell proliferation (Blangero and Teissie, 1985; Zhen et al., 2015). With prolonged contact time, the contact inhibition effect of hydrogels gradually showed. According to the results of live/dead staining, we chose Mg30, Mg20, Mg10, and Ctrl groups.

### Antibacterial Activity of the Hydrogels

**FIGURE 4:**
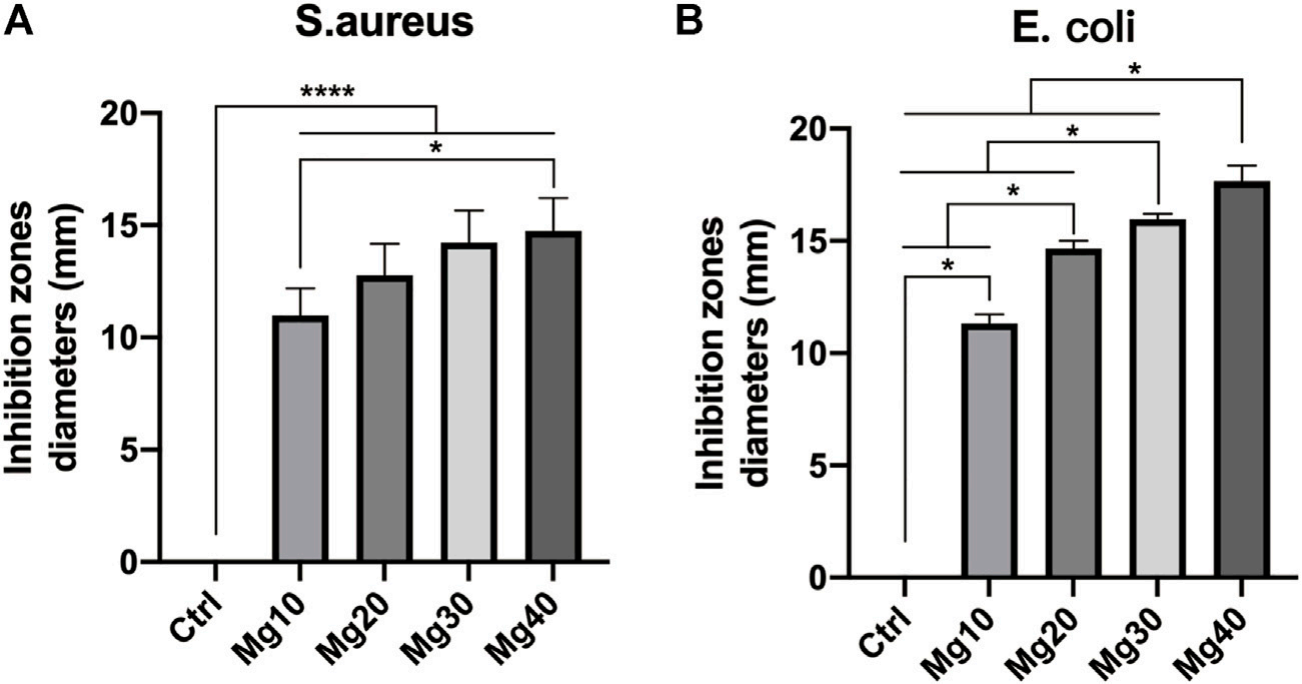
The antibacterial activity of MgSO_4_/MgO/SA/Na-CMC composite hydrogel dressings on Escherichia coli (ATCC 8739) and Staphylococcus aureus (ATCC 6538). P ≤ 0.05 was considered statistically significant. P ≤ 0.05 (*) P ≤ 0.0001(****).

Bacterial infection can seriously affect wound healing. The hydrogels prepared by us require anti-infection and antibacterial properties. We used two representative strains, *Escherichia coli* and *Staphylococcus aureus*, to test the antibacterial activity of hydrogels. The five groups of hydrogels were co-cultured with *Escherichia coli* and *Staphylococcus aureus* in a 37°C incubator. After 2 days of cultivation, the diameter of the inhibition zone was measured as follows: Mg40 > Mg30 > Mg20 > Mg10 > Ctrl (Figure 4). All of these proved that these hydrogels had good antibacterial activity, especially the Mg40 and Mg30 groups, showing great potential for antibacterial and anti-infection properties.

Reducing the risk of infection or antibacterial effect is one of the important conditions for evaluating wound dressings. Metal ions (such as silver ions) were often used in various skin dressings to inhibit the growth of bacteria (Tao et al., 2021). However, the role of silver ions was limited and could not alleviate the problem of surgical incision edema. Therefore, we chose Mg^2+^. As mentioned earlier, MgSO_4_ had good water absorption. Moreover, some researchers have reported that Mg^2+^ has antibacterial properties (Zhou et al., 2021). MgO is easily hydrated and forms a layer of magnesium hydroxide on the surface. A high concentration of reactive oxygen ions can exist on the surface of magnesium oxide. Reactive oxygen species ions are characterized by strong oxidation, which can destroy the peptide bond structure of bacterial cell membrane walls and quickly kill bacteria (Ikram et al., 2021). Therefore, the hydrogels prepared by us also showed antibacterial effects through antibacterial experiments. It is the best choice of dressing raw materials for postoperative surgical incision.

### Detumescence Effect of Hydrogels in an Edema Model

**FIGURE 5:**
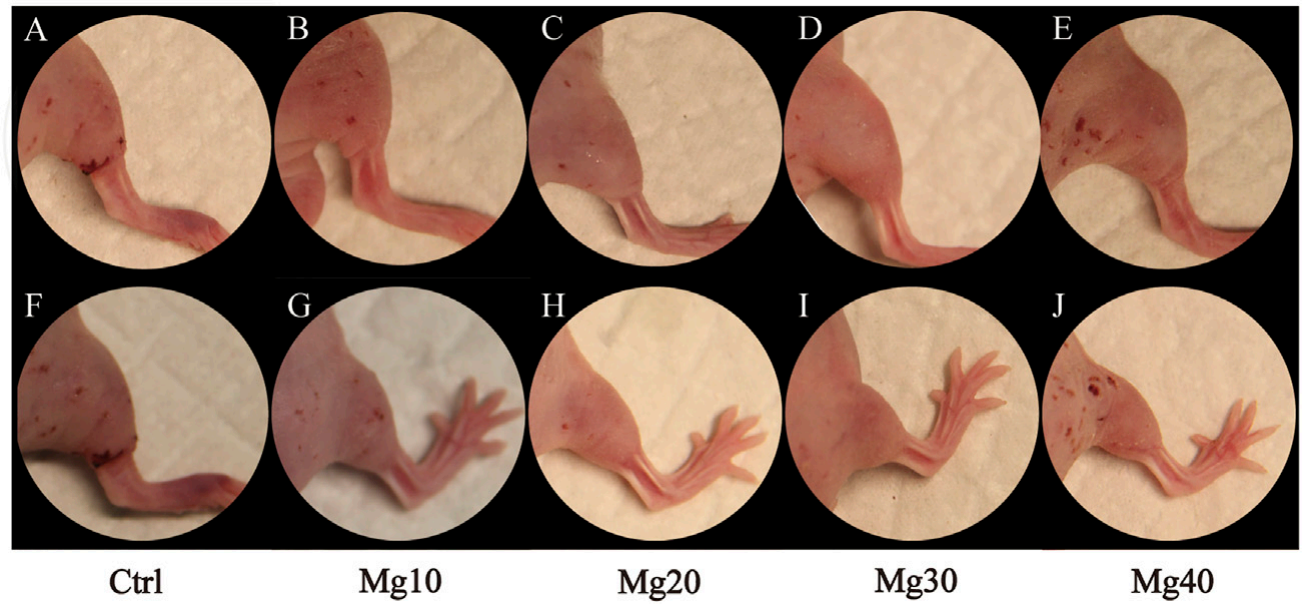
Hindlimb edema model in nude mice **(A–E)**, and after treatment of MgSO_4_/MgO/SA/Na-CMC composite hydrogel dressings **(F–J)**.

**FIGURE 6:**
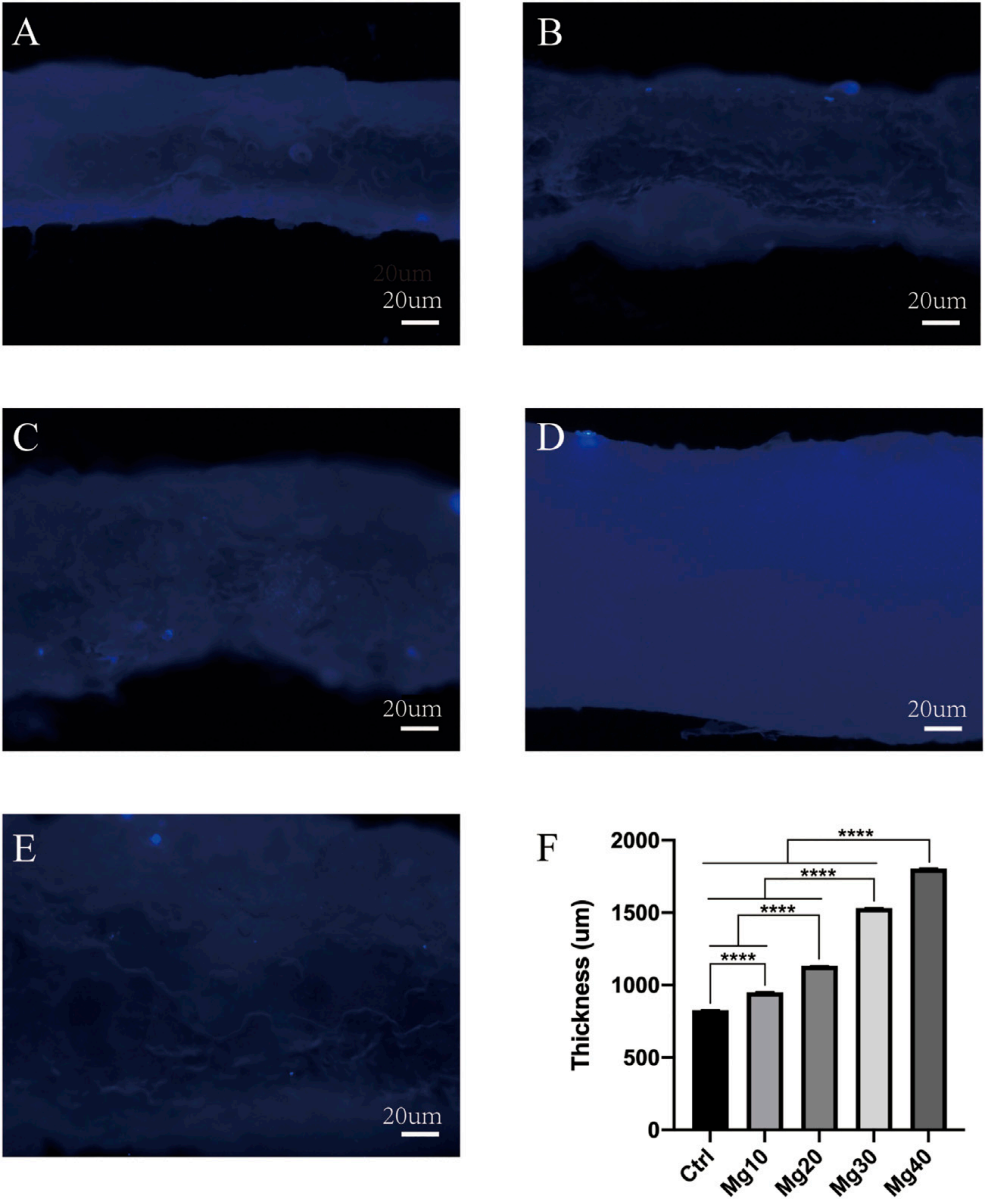
The water absorption of different groups (Ctrl, Mg10, Mg20, Mg30, Mg40) of MgSO_4_/MgO/SA/Na-CMC composite hydrogels under fluorescence microscope **(A–E)**. P ≤ 0.0001 was considered statistically significant (****).

After constructing the edema model in nude mice, the detumescence effect of the hydrogel was detected by *in vivo* tests. A visual examination showed that the extremity of edema in nude mice was obvious, and the hydrogels adhered to the incision of nude mice and were removed after 6–8 h. Figure 5 shows the contrast between the incision before and after the use of hydrogels. Visual examination between the groups showed that the detumescence effect of the four treatment groups was satisfactory, and Mg40 and Mg30 groups were significantly better than the other groups. Our hydrogels had an obvious effect on detumescence. During the experiment, neither infection nor rejection was observed, demonstrating that the hydrogels were biocompatible. The hydrogel was cut longitudinally and observed under a fluorescence microscope. It can be seen that the fluorescent bands of Mg40 and Mg30 were the widest. The order of the fluorescence bands from wide to narrow is Mg40 > Mg30 > Mg20 > Mg10 > Ctrl (Figure 6). This displayed that most water absorption was observed in the Mg40 and Mg30 groups. This signified that these two groups in the hydrogels had a better detumescence effect.

In the past decade, many studies were focused on the preparation of hydrogel dressing for wound healing and had achieved remarkable results (Cheng et al., 2021). These hydrogel dressings not only had antibacterial properties but also had many functions such as self-healing, hemostasis, and so on (Li et al., 2021). However, few researchers have noticed the impact of surgical incision edema on patients. We took full advantage of the hyperosmolar effect of MgSO_4_ and added it to the hydrogel scaffold to prepare MgSO_4_/MgO/SA/Na-CMC composite hydrogels. The model of postoperative incision edema in nude mice could show an obvious detumescence effect.

## Conclusion

MgSO_4_, MgO, SA, and Na-CMC were chosen as the main components of the composite hydrogel dressings. These materials were mixed according to the proportion of each group listed in Table 1, and a group was selected with good biocompatibility, antibacterial activity, and detumescence effect. According to the SR, we screened Mg40 and Mg30 groups. Biocompatibility suggested that the cell mortality of Mg40 was higher than that of the other four groups, and mechanical test results indicated that Mg40 has a significantly worse compression modulus than the other four groups, so Mg40 was excluded. In the antibacterial activity, it could be seen that Mg40 and Mg30 groups had greater inhibition on *Escherichia coli* and *Staphylococcus aureus*. In animal experiments, except for the Ctrl group, Mg10, Mg20, Mg30, and Mg40 all had strong detumescence ability. In conclusion, the Mg30 group not only showed excellent antibacterial activity and detumescence effect but also had good biocompatibility. We successfully prepared MgSO_4_/MgO/SA/Na-CMC composite hydrogel dressings with high water absorbency and antibacterial properties.

## Data Availability

The data that support the findings of this study are available on request from the corresponding author.
