# Supplementary material for: Highly Water-Absorptive and Antibacterial Hydrogel Dressings for Rapid Postoperative Detumescence
Source: Front Bioeng Biotechnol. 2022 May 13;10:845345. doi: 10.3389/fbioe.2022.845345 (PMC9136214; doi:10.3389/fbioe.2022.845345)
Supplement: Supplementary file 1 [file DataSheet1.docx]

Supplementary Material

## Supplementary Figures


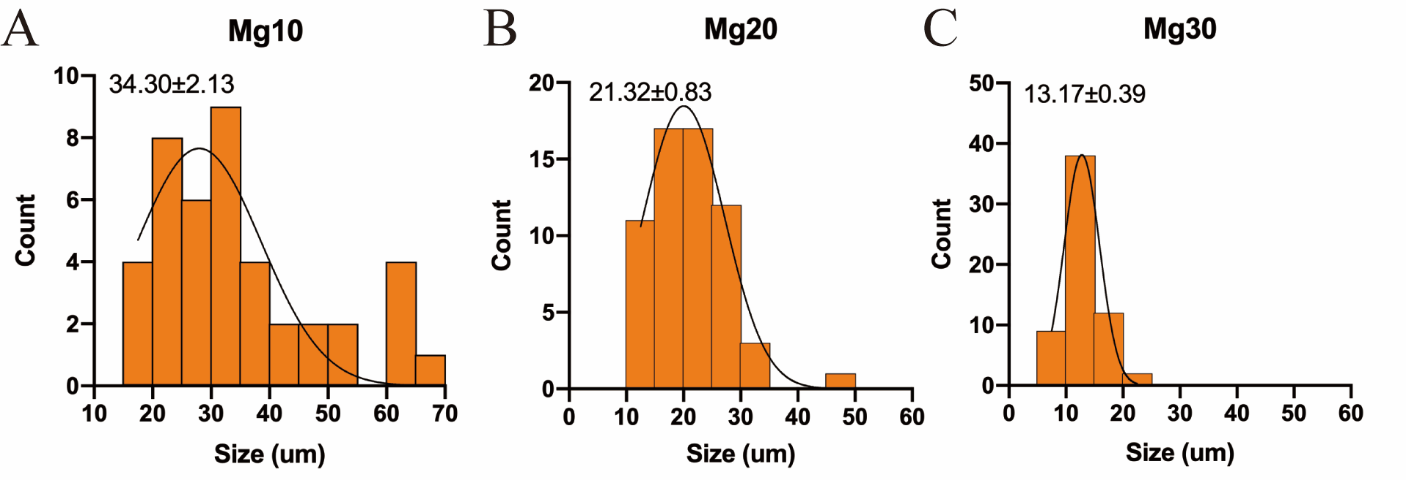


**Supplementary Figure 1.** The pore size distribution histogram of hydrogel (groups Mg10, Mg20, Mg30).


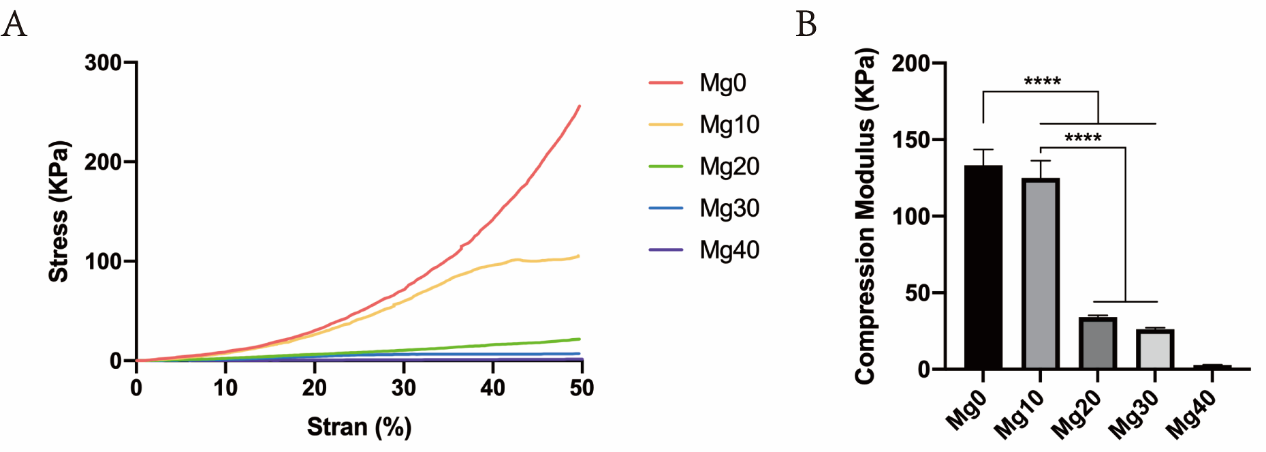


**Supplementary Figure 2.** The stress-strain curve (A) and compression modulus (B) of hydrogels (groups Mg10, Mg20, Mg30).
